# Recorrido y barreras en el acceso a diagnóstico y tratamiento de la enfermedad de Chagas en Argentina: un mapa desde la experiencia de las personas

**DOI:** 10.1590/0102-311XES251025

**Published:** 2026-10-26

**Authors:** Serena Eloisa Moyano, Catalina Canosa, Milagros Girart, Marina Gold, Carolina Lopez-Ferloni

**Affiliations:** 1 Fundación Mundo Sano, Buenos Aires, Argentina.; 2 Fundación Mundo Sano, Madrid, España.

**Keywords:** Enfermedad de Chagas, Enfermedades Desatendidas, Navegación de Pacientes, Barreras de Acceso a los Servicios de Salud, Chagas Disease, Neglected Diseases, Patient Navigation, Barriers to Access of Health Services, Doença de Chagas, Doenças Negligenciadas, Navegação de Pacientes, Barreiras ao Acesso aos Cuidados de Saúde

## Abstract

El Chagas es una enfermedad endémica en la región de las Américas. En la República Argentina hay aproximadamente 750.000 personas infectadas. Con una prevalencia de 1,59 cada 100 habitantes, se considera un problema de la salud pública. Este trabajo presenta la elaboración de un mapa de recorrido del paciente en personas con sospecha de Chagas. Se analizaron los registros de 205 consultantes en un programa de acompañamiento a personas con inquietudes sobre la enfermedad de Chagas en Argentina. La sistematización de experiencias personales mediante análisis cualitativo permite visibilizar los puntos de contacto críticos del paciente con el sistema de salud en el proceso de diagnóstico y tratamiento, identificando barreras de acceso al sistema de salud. La contribución principal del análisis es la demostración de la preponderancia de las barreras sistémicas sobre las personales como obstáculos para acceder al diagnóstico y sobre todo al tratamiento. La barrera más significativa resultó ser información incorrecta o insuficiente brindada por parte de los profesionales de salud, lo cual apunta a medidas concretas de capacitación a equipos de salud como estrategia de mejora de acceso al tratamiento.

## Abstract (en)

Chagas disease is endemic in the Americas. In Argentina, approximately 750,000 people are living with Chagas disease. With a prevalence of 1.59 per 100 inhabitants, it is considered a public health problem. This study presents the development of a patient journey map based on the experiences of individuals with suspected Chagas disease. We analyzed the records of 205 patients who sought care through a support program for individuals with concerns about Chagas disease in Argentina. By systematizing personal experiences through qualitative analysis, we were able to highlight the critical points of contact between patients and the healthcare system during the diagnosis and treatment process, thereby identifying barriers to accessing the healthcare services. The main contribution of this analysis is to demonstrate that systemic barriers outweigh personal barriers as obstacles to accessing diagnosis and, above all, treatment. The most significant barrier proved to be incorrect or insufficient information provided by healthcare professionals, which points to targeted training initiatives for healthcare teams as a strategy for improving access to treatment.

## Resumo

A doença de Chagas é endêmica na região das Américas. Na República Argentina, há aproximadamente 750 mil pessoas infectadas. Com uma prevalência de 1,59 por 100 habitantes, a doença é considerada um problema de saúde pública. Este trabalho apresenta a elaboração de um mapa do percurso clínico de pessoas com suspeita de doença de Chagas. Foram analisados os registros de 205 pacientes atendidos em um programa de acompanhamento a pessoas com questões sobre a doença de Chagas na Argentina. A sistematização das experiências pessoais por meio da análise qualitativa permite identificar os pontos críticos de contato do paciente com o sistema de saúde no processo de diagnóstico e tratamento, identificando barreiras de acesso ao sistema de saúde. A principal contribuição da análise é a demonstração da preponderância das barreiras sistêmicas sobre as pessoais como obstáculos ao acesso ao diagnóstico e, sobretudo, ao tratamento. A barreira mais significativa revelou-se ser as informações incorretas ou insuficientes fornecidas pelos profissionais de saúde, o que aponta para medidas concretas de capacitação das equipes de saúde como estratégia para melhorar o acesso ao tratamento.

## Introducción

El Chagas es una enfermedad endémica en la región de las Américas, producida por el parásito *Trypanosoma cruzi* y transmitida al ser humano principalmente por un insecto vector, el *Triatoma infestans*, comúnmente conocido en Argentina como vinchuca. Según datos de la Organización Mundial de la Salud (OMS) se estima que hay de 6 a 7 millones de infectados en todo el mundo ^1^. La enfermedad se transmite también de forma vertical durante el embarazo y el parto; oral, por consumo de alimentos contaminados y por transfusiones de sangre o donación de órganos infectados.

Actualmente, la enfermedad de Chagas se encuentra en el listado de enfermedades tropicales desatendidas de la Organización Panamericana de la Salud (OPS) ^2^, reportando más de 750.000 personas infectadas en la República Argentina y 6.243.803 expuestas al vector ^3^. Aunque se considera un problema de relevancia en el ámbito de la salud pública, con una prevalencia de 1,59 cada 100 habitantes, el 70% de quienes tienen Chagas no lo sabe, evidenciando un alto nivel de subdiagnóstico ^1^ ^,^ ^2^. Esto ocurre a pesar de existir el marco normativo necesario para establecer diagnóstico y tratamiento, y la disponibilidad de dos fármacos para su tratamiento. Este estudio cualitativo se focaliza en las barreras de acceso que encuentran las personas afectadas para obtener un diagnóstico apropiado y un tratamiento oportuno. A través de la realización de un mapa de recorrido de paciente ^4^ ^,^ ^5^ ^,^ ^6^ ^,^ ^7^, se analiza la complejidad de acceso desde la perspectiva de las personas que atraviesan el proceso de diagnóstico y tratamiento, para identificar barreras personales y estructurales que impactan en la capacidad del sistema de salud de captar apropiadamente a pacientes con enfermedad de Chagas.

## Marco regulatorio argentino

La República Argentina cuenta con un marco normativo para el abordaje de la enfermedad de Chagas, regulado principalmente por la *Ley 26.281*, sancionada en 2007 y reglamentada en 2022 ^8^. Otras normativas adicionales reglamentan el proceso de diagnóstico y tratamiento, como la *Ley 26.529* de Derechos del Paciente en su Relación con los Profesionales e Instituciones de la Salud ^9^, la *Ley 23.592* de Medidas contra Actos Discriminatorios ^10^ y la *Ley 15.465* de Enfermedades y Eventos de Notificación Obligatoria ^11^. Por ley, tanto el diagnóstico como el tratamiento de Chagas deben ser provistos en todos los hospitales y centros de salud de manera gratuita.

Existen guías nacionales, elaboradas por el Ministerio de Salud de la Nación, que estandarizan y detallan las técnicas que deben ser utilizadas para el diagnóstico y los medicamentos antiparasitarios recomendados junto al manejo clínico del paciente ^12^ ^,^ ^13^ ^,^ ^14^. Actualmente, se recomienda la realización de dos reacciones serológicas normatizadas de principios distintos para la confirmación de un diagnóstico ^12^; y se encuentran disponibles dos tratamientos etiológicos para el tratamiento de la enfermedad: el benznidazol y nifurtimox los cuales son distribuidos por el Programa Nacional de Chagas a las diferentes provincias que, a su vez, los entregan al sistema de salud provincial. También se encuentran disponibles la *Carta de Derechos en Chagas* ^15^ y los *Lineamientos Generales para el Abordaje Comunicacional de Chagas* ^16^, documentos que detallan y recomiendan el abordaje integral del paciente y sus derechos.

En Argentina, el sistema de atención de la salud abarca a toda la población del país a través de tres subsistemas que se diferencian en sus características normativas, organizativas, prestacionales y de financiación ^17^ ^,^ ^18^. El subsistema público comprende la red de hospitales y centros de atención estatal y de acceso gratuito que debiera brindar atención a toda persona que lo demande, principalmente a población sin otro tipo de cobertura. El subsistema de la Seguridad Social Obligatoria refiere a las distintas obras sociales que cubren a los trabajadores asalariados y a los jubilados, junto con sus familias. Por otro lado, el subsistema privado está compuesto por las entidades de medicina prepaga, profesionales de la salud independientes, clínicas, sanatorios y hospitales privados, cooperativas y mutuales de salud, a los que se accede mediante contratación directa o por derivación de aportes y contribuciones de la seguridad social.

El sistema de salud argentino es descentralizado en la gestión estatal, lo que requiere de articulación y colaboración de los gobiernos a nivel nacional, provincial y municipal. Asimismo, el área estatal se estructura en tres niveles de atención, clasificados según su capacidad de resolución y articulados mediante redes de servicios y un sistema de referencia-contrarreferencia, que otorga jerarquía a la atención primaria ^19^. Las guías nacionales establecen la prioridad de tratar la enfermedad de Chagas en el primer nivel de atención ^12^.

A pesar de existir legislación y documentos de trabajo que explicitan la necesidad de realizar diagnóstico y tratamiento a la población indicada, y la disponibilidad de pruebas de diagnóstico y de dos fármacos para el tratamiento, sigue habiendo sub-diagnóstico y falta de tratamiento de los afectados. Se estima que solo el 1% de quienes son diagnosticados reciben tratamiento ^20^. Un análisis más detallado de las experiencias de las personas para obtener diagnóstico y tratamiento permite identificar puntos débiles del sistema de salud y fortalecer estrategias de acceso.

## Marco teórico

Para analizar los factores involucrados en el acceso al diagnóstico y tratamiento de la enfermedad de Chagas, resulta fundamental considerar los determinantes sociales de la salud, definidos por la OMS ^21^ como “*las circunstancias en que las personas nacen, crecen, trabajan, viven y envejecen, y las fuerzas más amplias que conforman las condiciones de la vida cotidiana*”. Algunos ejemplos de estos determinantes sociales o aspectos no médicos de la salud incluyen educación, acceso a alimentos nutritivos, vivienda y trabajo dignos, inclusión social y no discriminación, acceso a servicios de salud asequibles, así como las condiciones socioeconómicas y políticas más amplias ^22^.

Desde 2009, la perspectiva de los determinantes sociales de la salud ha estado presente en las propuestas de la OMS, destacando los aspectos psicosociales, culturales y económico-políticos que afectan la salud, más allá de las perspectivas estrictamente biologicistas ^23^. Específicamente, el impacto que las enfermedades tropicales desatendidas tienen sobre el desarrollo de las comunidades está fuertemente atravesado por los determinantes sociales, entre los que destacan la pobreza, el acceso a agua y saneamiento, la vivienda, el medio ambiente y factores socioculturales, entre otros ^24^.

Asimismo, Gold et al. ^25^ señalan que los determinantes sociales de la salud están jerarquizados e influidos por estructuras de poder y, al mismo tiempo, por factores a nivel micro de las relaciones sociales (como las contingencias personales, las redes sociales o las concepciones culturales). De esta forma, ambas dimensiones influyen en la capacidad de atención de los sistemas de salud y en las actitudes de las personas hacia dichos sistemas.

Los determinantes sociales pueden tanto realzar la salud como afectarla negativamente, contribuyendo a reforzar barreras de acceso a diferentes instancias de la atención médica. Adicionalmente, como argumentan Griese et al. ^26^, la expansión de la especialización médica resulta en sistemas cada vez más complejos y confusos para los usuarios, requiriendo que estos identifiquen cómo ingresar y qué recorridos atravesar para resolver los problemas que se les presentan a nivel sanitario. El diagnóstico y tratamiento de la enfermedad de Chagas se ve afectado por la complejidad de los sistemas de salud, al requerir intervenciones de múltiples especialistas (cardiología, infectología, pediatría, ginecología, atención primaria), y al afectar a poblaciones más vulnerables a los determinantes sociales de salud.

Para visibilizar la complejidad de un diagnóstico oportuno y acceso a tratamiento de personas afectadas por la enfermedad de Chagas, se recurre a la metodología de mapa de recorrido del paciente, en inglés *patient journey map*, que permite resaltar cualitativa y cuantitativamente los puntos críticos en la interacción de pacientes con el sistema de salud. Un mapa de recorrido del paciente es una herramienta visual, originalmente ideada en el campo del *marketing*, que permite graficar la experiencia que un usuario atraviesa en un determinado proceso. Aplicado al campo de la salud, posibilita entender cuáles son los puntos críticos de contacto con el sistema de salud de un paciente en relación con un curso de atención específico ^4^ ^,^ ^5^ ^,^ ^6^ ^,^ ^7^.

El objetivo de estos mapas, en el campo de la salud, es entender qué siente, piensa y experimenta una persona que recorre un proceso de atención médica. En este trabajo buscamos abordar la problemática de Chagas desde el punto de vista de las personas que atraviesan el proceso de diagnóstico y tratamiento de la enfermedad.

Existen trabajos, entre los cuales se encuentra la hoja de ruta de la enfermedad de Chagas de la OMS ^27^ que presenta un análisis sobre los obstáculos de acceso a un cuidado integral de los pacientes con Chagas, proponiendo estrategias y soluciones para profesionales de la salud, autoridades sanitarias y gobiernos para ayudar a superar las barreras para el cuidado integral de los pacientes. En este trabajo se destaca el conocimiento insuficiente y una subestimación de la importancia del diagnóstico y tratamiento temprano por parte de las comunidades médicas como la barrera principal de acceso.

La herramienta de mapa de recorrido del paciente nos permite comprender cuáles son las necesidades del paciente desde su propia experiencia, así como las dificultades identificadas en su propio proceso de atención, aportando información que pueda resultar útil para abordar la problemática de acceso a la salud. En temas complejos como la enfermedad de Chagas que requieren abordaje médico y de salud pública integrales, una herramienta como esta nos permite “ponernos en los zapatos” de las personas afectadas e identificar las barreras existentes y poder derribarlas, sobre la base de evidencia científica.

## Metodología

Fundación Mundo Sano realiza desde 2019 acompañamiento a personas con inquietudes sobre la enfermedad de Chagas, que, a fines de este trabajo, denominaremos “personas consultantes”. Esta fundación sin fines de lucro, fundada en 1993, realiza investigación interdisciplinaria e implementación en temáticas de salud que afectan a comunidades desatendidas. A mediados de 2019, ante la demanda de personas que se comunicaban con la organización para pedir información sobre diagnóstico y tratamiento de Chagas, y teniendo en cuenta la experiencia en la temática, se buscó dar respuesta a estas inquietudes de forma institucional.

El principal objetivo de este acompañamiento es brindar asesoramiento sobre diagnóstico y/o tratamiento de Chagas, durante el tiempo que dure dicho proceso. Se lleva a cabo de forma telefónica, por un equipo de tres personas, y se sistematizan los casos en planillas de cálculo, registrando cada uno de los contactos con la persona. Durante las conversaciones de seguimiento se relevan datos demográficos, motivos de consulta, inquietudes de la persona y el estatus de diagnóstico y/o tratamiento, en base a un procedimiento estándar de llamado y registro de datos.

Tomando como punto de partida los registros periódicos de acompañamiento, se realizó un análisis retrospectivo de 205 casos correspondientes al período 2019-2024. Los mismos representan la totalidad de los casos acompañados por la organización en dicho período y fueron anonimizados para proteger la identidad de las personas, con lo cual no se contó con criterios de exclusión. La duración del proceso de acompañamiento en cada uno de los casos es particular y atiende a las necesidades de la persona consultante, sus acciones y su decisión de continuar o no con el proceso, por lo cual no hay un tiempo de acompañamiento estándar. Algunos casos son acompañados en todo su proceso de diagnóstico y tratamiento y otros solo durante alguna etapa.

A partir de estos datos, se llevó a cabo un proceso iterativo, enmarcado en instancias de discusión periódica del equipo de trabajo, de identificación y codificación de categorías, en función de los patrones que iban emergiendo de los relatos de los consultantes, que incluyó una etapa de codificación y revisión ciega. Los datos analizados tienen en cuenta tanto experiencias de los pacientes, acontecidas durante el proceso de acompañamiento, como también aquellas que habían sucedido anteriormente y fueron reportadas en el registro del caso. En todo momento se toma como fuente de información los relatos provistos por las personas acompañadas, el equipo de acompañamiento no tuvo interacción con los profesionales de salud que atendieron a los consultantes.

Con la información codificada, se identificaron y definieron los puntos de contacto de la persona con el sistema de salud en el proceso de diagnóstico y tratamiento de la enfermedad de Chagas, y se generaron categorías de etapas y barreras existentes a partir de la agregación de dicha información.

Las barreras fueron cuantificadas en función de la cantidad de veces que dicha problemática aparecía en los textuales de los consultantes, en cada una de las etapas, en los registros de acompañamiento. Una persona puede haber experimentado todas las barreras en todas las etapas, haber experimentado solo algunas o haber referido no experimentar ninguna. Del mismo modo, los diferentes consultantes pueden atravesar una, varias o todas las etapas en su proceso de acompañamiento, dependiendo del momento en que se hayan contactado con la Fundación Mundo Sano para solicitar el mismo.

Al encontrar barreras similares en las diferentes etapas, se ha decidido mantener el mismo nombre y explicitar cómo se manifiestan en dicha etapa, brindando ejemplos que han expresado los consultantes.

## Resultados y discusión

De los 205 consultantes que ingresaron al proceso de acompañamiento, 114 solicitaron información sobre cómo realizar o confirmar su diagnóstico al equipo de acompañamiento. En la Tabla 1 se detallan las características sociodemográficas de los consultantes. Teniendo en cuenta el ya mencionado elevado índice de sub-diagnóstico de la enfermedad, este número resulta especialmente relevante.

**Tabla 1:** Características sociodemográficas de los consultantes.

Variable	n	% *
Género		
Femenino	156	76,1
Masculino	49	23,9
Edad (años)		
18-40	63	30,7
41-50	55	26,8
51-55	19	9,3
+ 56	49	23,9
Sin información	19	9,3
Lugar de nacimiento		
Argentina	102	49,8
Santiago del Estero	26	12,7
Buenos Aires	14	6,8
Chaco	13	6,3
Salta	11	5,4
Ciudad Autónoma de Buenos Aires	9	4,4
Jujuy	6	2,9
Misiones	5	2,4
Mendoza	4	2,0
Tucumán	4	2,0
Córdoba	3	1,5
Formosa	3	1,5
Catamarca	2	1,0
Santa Fe	1	0,5
Chubut	1	0,5
Bolivia	68	33,2
Paraguay	9	4,4
México	1	0,5
Brasil	1	0,5
Sin información	24	11,7
Lugar de residencia actual		
Argentina	200	97,6
Buenos Aires	139	67,8
Ciudad Autónoma de Buenos Aires	32	15,6
Jujuy	7	3,4
Salta	6	2,9
Córdoba	6	2,9
Santiago del Estero	4	2,0
Misiones	2	1,0
Mendoza	2	1,0
Tucumán	1	0,5
Santa Fe	1	0,5
México	1	0,5
Colombia	1	0,5
Israel	1	0,5
Sin información	2	1,0
Fuente de sospecha de Chagas		
Análisis durante el embarazo	63	30,7
Análisis durante la donación de sangre	29	14,1
Análisis de ingreso a institución	17	8,3
Control clínico	15	7,3
Aparición de síntomas	7	3,4
Sospecha por familiar con Chagas	6	2,9
Sensibilización por medios de comunicación	1	0,5
Sin información	67	32,7

* Los porcentajes fueron redondeados para clarificar la lectura.

Al momento de finalización del análisis de datos, 54 (26,3%) contaban con un diagnóstico confirmado, realizado por dos pruebas serológicas diferentes, como lo indican las guías nacionales -42 (20,5%) resultaron positivos y 12 (5,8%) negativos, de los cuales 38 (18,5%) fueron acompañados por la Fundación Mundo Sano a lo largo del proceso diagnóstico. Con diagnóstico positivo previo, se identificaron 128 (62,4%) personas, y 3 (1,5%) tenían un diagnóstico negativo, pero no contaban con la documentación que respaldara el diagnóstico o bien los mismos no habían sido realizados acorde a las recomendaciones de las guías nacionales. Al momento de cierre de este trabajo, aproximadamente la mitad de los casos se encontraban cerrados y la otra mitad continuaba en proceso de acompañamiento.

### Mapa de recorrido del paciente de personas con sospecha de Chagas

Se definieron cinco momentos en que las personas consultantes entraban en contacto con el sistema de salud en su proceso de diagnóstico y tratamiento: (0) Desconocimiento, (1) Diagnóstico confirmatorio, (2) Inicio del tratamiento, (3) Finalización del tratamiento y (4) Controles periódicos (Tabla 2; Figura 1). La duración de cada una de ellas es variable y depende del recorrido particular de cada consultante. Los pasos de una etapa a la otra representan momentos críticos donde se puede avanzar, estancarse o abandonar el proceso. En cada una de las etapas, el sistema de salud brinda información y herramientas para avanzar a la siguiente. Es en estos momentos de inflexión donde se identificaron y agruparon los obstáculos referidos por las personas, como barreras en el recorrido, que impiden la continuidad del proceso.

**Tabla 2:** Resumen de las barreras detectadas a lo largo del recorrido de las personas acompañadas durante las diferentes etapas en el nivel del sistema de salud.

Etapa	Barrera			
	Información incorrecta o insuficiente por parte del equipo de salud	Dificultad o imposibilidad para acceder a los estudios o exámenes clínicos solicitados por el profesional de salud	Dificultad para acceder o completar el tratamiento	Dificultades en el recorrido del circuito de atención
0 - Desconocimiento	14	8	-	6
1 - Diagnóstico confirmatorio	75	9	13	10
2 - Inicio del tratamiento	1	-	17	1
3 - Finalización del tratamiento	4	12	-	3
Total	94	29	30	20

**Figura 1:**
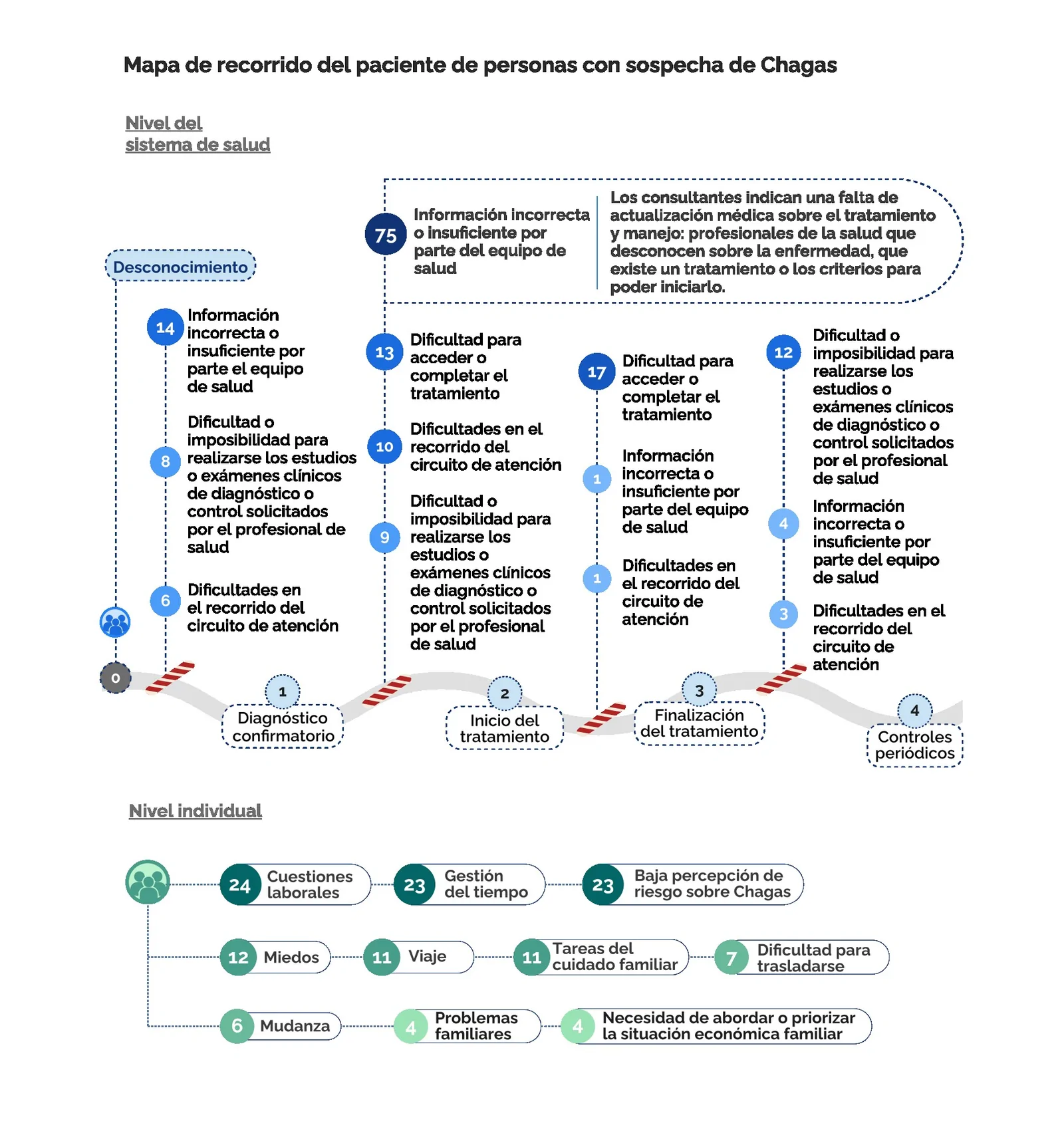
Mapa de recorrido del paciente de la persona con sospecha de Chagas en Argentina, con el resumen ilustrativo de sus componentes, construido en base a los relatos de las 205 personas consultantes y su experiencia con el diagnóstico y tratamiento de la enfermedad de Chagas.

En cada una de las etapas se identificaron diferentes barreras de acceso al diagnóstico y tratamiento de la enfermedad de Chagas. Para poder entender el recorrido de las personas a lo largo del proceso e identificar los puntos a mejorar durante la atención de la persona, se ordenaron los elementos relevados en dos niveles: individual y el del sistema de salud. Si bien se establecieron las barreras agrupadas gráficamente (Figura 1) para mayor claridad, estas pueden suceder en cualquier momento durante esa etapa.

Los niveles se dividieron en dos principales categorías a fines de comprender una problemática multidimensional, respondiendo a dos grandes agrupaciones de problemas: aquellos que dependen de la experiencia personal de la persona, y aquellos que se refieren específicamente al sistema de salud. En el nivel individual, se incluyeron aspectos individuales de la experiencia del paciente, e involucra tanto factores personales que inciden en el recorrido (como sus emociones, creencias y actitudes) como los determinantes sociales de la salud (contexto socioeconómico y político, las condiciones de empleo y trabajo, la vivienda, el transporte, las condiciones psicosociales). El nivel del sistema de salud refiere al conjunto de personas, instituciones y recursos que proveen asistencia sanitaria, con el objetivo de mantener y mejorar la salud de la población, y permite entender cuáles son los obstáculos identificados por las personas que lo atraviesan. Al ser un estudio que se basa en las experiencias de las personas, la caracterización de las barreras del sistema de salud está construida en función de estas subjetividades.

Durante todo el recorrido de la persona acompañada, se identificaron tres barreras constantes en el nivel del sistema de salud: información incorrecta o insuficiente por parte del equipo de salud sobre Chagas, dificultad o imposibilidad para realizarse los estudios o exámenes clínicos de diagnóstico o control solicitados por el profesional de salud, y dificultades en el recorrido del circuito de atención. A su vez, durante el recorrido, se identificó una cuarta barrera, exclusivamente en las etapas 2 y 3: dificultad para acceder o completar el tratamiento.

### Etapa 0: desconocimiento de la enfermedad

Este es el punto de partida en el recorrido de una persona que sospecha que puede tener la enfermedad de Chagas, pero no tiene aún diagnóstico certero. Se han identificado dos posibles causas principales: falta de acceso al sistema de salud donde la persona no ha realizado una consulta médica o no se le ha ofrecido el diagnóstico de Chagas en las mismas; o falta de información fehaciente sobre la enfermedad debido al desconocimiento, ya sea de la enfermedad, sus formas de transmisión o riesgos para la salud. Otra falta de información tiene que ver con conocimiento limitado, por ejemplo, en casos donde la persona ha escuchado hablar de la enfermedad o tiene algún nivel de conocimiento de la problemática por residir en zona endémica o conocer allegados o familiares con la enfermedad, entre otros motivos, pero no ha realizado un diagnóstico propio ni recibido información suficiente para identificarse como en riesgo de contraer Chagas. En estos casos se evidencia un sistema de salud que ha fallado en proveer la información necesaria a los pacientes.

En esta etapa se produce el ingreso al sistema de salud. Durante el primer contacto con el mismo, emerge la posibilidad de recibir un diagnóstico y, eventualmente, el tratamiento en caso de que correspondiese. Los consultantes identifican las siguientes vías de ingreso al sistema de salud:

• Análisis de rutina durante el seguimiento de un embarazo.

• Análisis de rutina de ingreso a institución (escuela/universidad/trabajo).

• Consulta médica por presencia de síntomas.

• Tamizaje ante donación de sangre.

• Control clínico: el médico indica como control o análisis de rutina la prueba de Chagas.

• Consulta por antecedentes de familiares con Chagas.

En esta primera etapa, se identificaron las barreras detalladas en la Tabla 3. La barrera más común (n = 14) fue la falta de información correcta o suficiente por parte del equipo de salud.

**Tabla 3:** Barreras del sistema de salud identificadas en la etapa 0 (desconocimiento de la enfermedad). Se detallan las barreras, su frecuencia y como se manifiestan.

Barreras del sistema de salud	Cómo se manifiestan en esta etapa	n
Información incorrecta o insuficiente por parte del equipo de salud	Los consultantes indican que el personal de salud no les ha brindado la orden médica para realizar el diagnóstico ni lo sugiere, aun encontrándose dentro de los grupos priorizados por la Ley de Chagas. También refieren que no se comunica adecuadamente el resultado del diagnóstico ni se sensibiliza sobre la enfermedad, acorde a la Ley de derechos del paciente	14
Dificultad o imposibilidad para brindar estudios clínicos de diagnóstico o control solicitados por el profesional de salud	Imposibilidad de realizar la prueba de Chagas: los consultantes refieren haberse encontrado con limitaciones en la infraestructura sanitaria, como la falta de reactivos o turnos disponibles para la atención	8
Dificultades en el recorrido del circuito de atención	Los consultantes refieren haber tenido dificultades para acceder a su historia clínica y experimentaron falta de respuesta por parte del sistema de salud en la búsqueda de turnos con profesionales médicos	6

Klein et al. ^28^ encuentran la falta de búsqueda sistemática de casos y la falta de capacitación de los profesionales de la salud como elementos clave en el subdiagnóstico de la enfermedad.

### Etapa 1: diagnóstico confirmatorio

En Argentina, acorde a las guías nacionales, se deben realizar al menos dos pruebas serológicas diferentes para confirmar o descartar la infección por *T. cruzi*. Este proceso es, por ley, gratuito y debe realizarse en hospitales y centros de salud. Además, no basta con confirmar el diagnóstico, es necesario que la persona entienda qué implica tener Chagas, cuáles son los beneficios de realizar el tratamiento, cuáles son sus efectos secundarios, qué opciones de tratamiento existen, y qué pasos debe seguir para avanzar en su recorrido. Durante esta etapa, el paciente busca atención médica adecuada, la cual debería recibir. Esta atención debería incluir información clara y fehaciente por parte del equipo de salud. En el caso de que el diagnóstico sea positivo para Chagas, esta instancia representa la posibilidad de acceder al tratamiento en la etapa siguiente, ya que es el momento donde se le comunica o no la existencia y disponibilidad de tratamiento etiológico.

Durante esta etapa, también se identificaron diferentes barreras (Tabla 4); la más frecuente (n = 75) está relacionada con la falta de información por parte de los equipos de salud. La información incorrecta o insuficiente en este caso refiere principalmente a la omisión de la existencia de tratamientos etiológicos durante la consulta o datos contradictorios con las guías nacionales.

**Tabla 4:** Barreras del sistema de salud identificadas en la etapa 1 (diagnóstico confirmatorio). Se detallan las barreras, su frecuencia y cómo se manifiestan.

Barreras del sistema de salud	Cómo se manifiestan en esta etapa	n
Información incorrecta o insuficiente por parte del equipo de salud	Los consultantes indican una falta de actualización médica sobre el tratamiento y su manejo apropiado: profesionales de la salud que desconocen sobre la enfermedad, que existe un tratamiento o los criterios para poder iniciarlo	75
Dificultad o imposibilidad para brindar estudios o exámenes clínicos de diagnóstico o control solicitados por el profesional de salud	Imposibilidad de realizar estudios complementarios (como electrocardiogramas, radiografías o colonoscopías) necesarios para determinar si la persona cumple con los criterios para iniciar el tratamiento. Los consultantes mencionan la dificultad para conseguir turnos, falta de orden médica correspondiente y derivaciones a otros efectores	9
Dificultad para acceder o completar el tratamiento	Falta de acceso al tratamiento. Los consultantes refieren que se les dificulta conseguir la medicación necesaria para el tratamiento en los centros de salud. Asimismo, mencionan la dificultad para conseguir turnos con profesionales especializados	13
Dificultades en el recorrido del circuito de atención	Los consultantes refieren haber encontrado obstáculos en el circuito de atención, relacionados con la falta de claridad en el mismo (dónde solicitar turnos o con qué profesional acudir), especialmente luego de derivaciones imprecisas o ante la ausencia de profesionales al momento de la consulta. También en esta instancia se indica haber experimentado falta de respuesta por parte del sistema a la hora de solicitar turnos	10

En este sentido, en Echeverría et al. ^27^ se identifica el conocimiento inadecuado como una barrera en el acceso al tratamiento de Chagas y se insta a los sistemas y profesionales de la salud a intensificar los esfuerzos para erradicar la enfermedad.

En la barrera “dificultad para acceder o completar el tratamiento”, si bien los consultantes refieren una dificultad para acceder a la medicación sin especificar su causa, la práctica a lo largo de los años nos ha mostrado que el tratamiento etiológico se encuentra disponible desde el punto de vista de su producción y las dificultades suelen residir en el proceso de distribución del mismo dentro de los diferentes niveles del estado (programa nacional, provincial, regiones sanitarias y centros de salud).

### Etapa 2: inicio del tratamiento

La persona inicia el tratamiento médico para la enfermedad de Chagas. El objetivo en esta etapa es no solo que la persona comience el tratamiento, sino también garantizar que tenga las condiciones necesarias para finalizarlo. Durante esta etapa se deben dar las condiciones, información y herramientas para dar continuidad al tratamiento hasta su finalización. El paso intermedio en esta etapa implica brindar al paciente herramientas y soporte continuo para que pueda completar el tratamiento. Esto requiere no solo de un adecuado acompañamiento médico, sino también de una infraestructura de salud que facilite la continuidad de los controles clínicos y la gestión de efectos adversos que puedan surgir durante el tratamiento. Las barreras detectadas durante esta etapa se detallan en la Tabla 5. En este caso, la barrera más frecuentemente detectada fue la dificultad para acceder o completar el tratamiento (n = 17).

**Tabla 5:** Barreras del sistema de salud identificadas en la etapa 2 (inicio del tratamiento).

Barreras del sistema de salud	Cómo se manifiestan en esta etapa y transición	n
Información incorrecta o insuficiente por parte del equipo de salud	Los consultantes indican que, en el pasado, los profesionales de la salud no les explicaron de forma clara las reacciones adversas experimentadas	1
Dificultad para acceder o completar el tratamiento	Aparición de reacciones adversas. Los consultantes indican la suspensión del tratamiento, el cual luego no se retoma	17
Dificultades en el recorrido del circuito de atención	Los consultantes refieren haber experimentado falta de respuesta por parte del sistema de salud	1

### Etapa 3: finalización de tratamiento

En esta etapa, la persona logra finalizar el tratamiento etiológico para tratar la infección por *T. cruzi*. En algunos casos el tratamiento fue completado, y en otros el paciente interrumpe el proceso debido a barreras o complicaciones. Sin embargo, este momento no significa el fin del recorrido, ya que el control y seguimiento regular son esenciales para garantizar el bienestar a largo plazo y prevenir complicaciones asociadas a la enfermedad. Durante esta etapa se brinda a la persona la información para continuar con controles regulares tras la finalización del tratamiento y se debe garantizar que:

• Acceda a controles clínicos periódicos.

• Obtenga información clara sobre cómo monitorear su salud.

• Pueda extender el conocimiento a su entorno familiar (si hay familiares en riesgo).

Este paso es crítico porque permite mantener el contacto entre la persona y el sistema de salud, favoreciendo un monitoreo constante y el acceso a tratamiento oportuno en caso de complicaciones. En esta etapa se detectaron tres barreras, la más frecuente (n = 12) se relaciona con la dificultad de acceder a controles clínicos regulares (Tabla 6).

**Tabla 6:** Barreras del sistema de salud identificadas en la etapa 3 (finalización del tratamiento).

Barreras del sistema de salud identificadas en esta etapa	Cómo se manifiestan en esta etapa	n
Información incorrecta o insuficiente por parte del equipo de salud	Los consultantes que finalizan el tratamiento o no cumplen los criterios para realizarlo manifiestan no tener claridad sobre los pasos subsiguientes: (i) Cómo controlar su salud de manera regular; (ii) Qué cuidados necesita para evitar complicaciones futuras	4
Dificultad o imposibilidad para brindar estudios o exámenes clínicos de diagnóstico o control solicitados por el profesional de salud	Imposibilidad de acceder a controles clínicos regulares como análisis de sangre o electrocardiogramas. Los consultantes refieren haberse enfrentado a limitaciones logísticas (falta de turnos o centros cercanos al domicilio)	12
Dificultades en el recorrido del circuito de atención	Los consultantes refieren haber tenido dificultades para acceder a su historia clínica y experimentaron falta de claridad en relación con el circuito de atención médica	3

### Etapa 4: controles periódicos

Esta etapa involucra controles, generalmente anuales, y el monitoreo del estado de salud. Las barreras presentes en esta etapa son las mismas que se identifican en la etapa 3 (Tabla 6). Es importante destacar que estos controles, en la mayoría de los casos, deben llevarse a cabo a lo largo de toda la vida de la persona.

En cuanto a las barreras a nivel individual, se establecieron de forma transversal en todas las etapas, ya que se presentan a lo largo de todo el recorrido que la persona atraviesa respecto a su salud. Algunas de ellas son más acotadas en el tiempo (como una mudanza o migración) y otras son más sostenidas (como un bajo nivel educativo o socioeconómico). En la Tabla 7 se pueden visualizar las principales barreras a nivel individual indicadas por los consultantes. Las barreras más frecuentes se relacionan con cuestiones laborales (n = 26), falta de tiempo (n = 23), y otros problemas de salud (n = 22). No todas las personas acompañadas presentaron barreras individuales y algunas personas presentaron más de una.

**Tabla 7:** Principales barreras identificadas a nivel individual durante el recorrido de las personas acompañadas a lo largo de todas las etapas.

Barreras a nivel individual	n
Cuestiones laborales: condiciones laborales rígidas que impiden que las personas puedan ir a las consultas médicas en horarios laborales, realizar el diagnóstico o tratamiento	24
Gestión del tiempo: falta de tiempo y dificultades de organización	23
Baja percepción de riesgo sobre Chagas: priorización de la atención de otras condiciones médicas	23
Tareas de cuidado familiar: personas que tienen a su cargo el cuidado de sus hijos principalmente y deben priorizar dichas tareas frente a su propia salud	11
Miedos: miedo a los efectos adversos, o a vivir experiencias por las que ya pasaron sus familiares (síntomas o fallecimientos)	12
Dificultad para trasladarse: lejanía de centros de salud o dificultad de acceso	7
Necesidad de abordar o priorizar la situación económica familiar	4
Problemas familiares: necesidad de abordar situaciones de conflicto dentro del ambiente familiar	4
Mudanza: cambio de domicilio que afecta la continuidad del recorrido	6
Viaje: viajes que surgen de manera urgente e impiden que sigan realizando los controles	11

Trabajos como el de Navarro et al. ^29^ plantean que las razones no solo biomédicas, sino sociales, intervienen en el proceso de diagnóstico y tratamiento y destacan la necesidad de abordajes que tengan en cuenta los aspectos culturales y económicos, entre otros. Gómez i Prat et al. ^30^, por su parte, identifican que un abordaje comunitario a través de promotores de la salud puede favorecer la reducción de barreras como el miedo y la desconfianza de los pacientes en el sistema de salud.

A pesar de las barreras personales, que no dejan de ser significativas, el resultado más sorprendente y contundente del estudio es la predominancia de las barreras dentro del sistema de salud, y sobre todo el desconocimiento del personal de salud, como barrera más significativa en todas las etapas del recorrido del paciente por intentar diagnosticar y tratar su problema. Esto genera una llamada a la acción clara, en cuanto a acciones de salud pública en torno a la enfermedad de Chagas, de fortalecer el sistema de atención primaria, desde el cual debiera poderse dar una respuesta más eficiente a personas con sospecha de infección por *T. cruzi*, sobre todo en lo que respecta a la prevención.

## Conclusiones

La problemática de la enfermedad de Chagas representa un desafío para los sistemas de salud. Es importante tener en cuenta el punto de vista de las personas que atraviesan el proceso de diagnóstico y tratamiento de Chagas, para poder comprender la forma en que las barreras dificultan cada una de las instancias de acceso al diagnóstico y tratamiento.

Teniendo en cuenta que la principal barrera identificada tiene que ver con la falta de información fehaciente o información insuficiente por parte del sistema de salud, resulta pertinente que futuros estudios colaboren con la construcción de conocimiento sobre cuáles son las principales creencias y conocimientos de los profesionales de la salud sobre la problemática de Chagas y, cómo estos se relacionan con las barreras identificadas por este y otros trabajos sobre el tema.

Hoy en día, existiendo en Argentina un marco regulatorio nacional con su correspondiente adhesión provincial, tratamientos etiológicos disponibles y guías nacionales de diagnóstico y tratamiento, los pacientes siguen encontrando la principal barrera en la falta de conocimiento por parte del sistema de salud y sus profesionales. Sin tener la intención de demonizar a los profesionales de salud, que todos los días intentan, entre recortes y desabastecimiento de recursos, realizar sus tareas y brindar atención y salud a las personas, intentamos que los datos recolectados en este estudio sirvan de evidencia para desarrollar programas de salud pública eficientes y focalizados a los espacios donde se puede generar una diferencia importante en atender a lo que se considera una enfermedad desatendida. Es necesario un abordaje integrado y multisectorial, que tenga en cuenta la formación de los profesionales de salud y el cumplimiento de los derechos de los habitantes a atención médica de calidad.

## Data Availability

Los datos de la investigación están disponibles previa solicitud a la autora correspondiente.
